# Ecological validity of virtual environments to assess human navigation ability

**DOI:** 10.3389/fpsyg.2015.00637

**Published:** 2015-05-27

**Authors:** Ineke J. M. van der Ham, Annemarie M. E. Faber, Matthijs Venselaar, Marc J. van Kreveld, Maarten Löffler

**Affiliations:** ^1^Department of Experimental Psychology, Utrecht University, Utrecht, Netherlands; ^2^Department of Information and Computing Sciences, Utrecht University, Utrecht, Netherlands

**Keywords:** navigation, virtual reality, route knowledge, survey knowledge, ecological validity

## Abstract

Route memory is frequently assessed in virtual environments. These environments can be presented in a fully controlled manner and are easy to use. Yet they lack the physical involvement that participants have when navigating real environments. For some aspects of route memory this may result in reduced performance in virtual environments. We assessed route memory performance in four different environments: real, virtual, virtual with directional information (compass), and hybrid. In the hybrid environment, participants walked the route outside on an open field, while all route information (i.e., path, landmarks) was shown simultaneously on a handheld tablet computer. Results indicate that performance in the real life environment was better than in the virtual conditions for tasks relying on survey knowledge, like pointing to start and end point, and map drawing. Performance in the hybrid condition however, hardly differed from real life performance. Performance in the virtual environment did not benefit from directional information. Given these findings, the hybrid condition may offer the best of both worlds: the performance level is comparable to that of real life for route memory, yet it offers full control of visual input during route learning.

## Introduction

Whenever we move around, whether it is from our bedroom to the kitchen or from our home to a foreign city, we rely on our navigation ability. For instance, we think about the location of our goal and our starting point, consider the landmarks we may encounter along the way, and may create mental maps of our environment to find the shortest route. Navigation ability has been studied extensively over the past few decades and still is a quickly developing research topic (for a review see, e.g., Wolbers and Hegarty, 2010). One important factor in these developments is the rapid improvement of technology, particularly pertaining to virtual environments. With these virtual environments navigation ability can be studied in any possible type of environment, and responses can be recorded in great detail. Yet, there may also be some disadvantages in comparison to testing in a real environment. The validity of using virtual reality (VR) in testing navigation ability is therefore still a matter of debate. In the current paper, we address this debate by directly comparing navigation performance in both real and different types of virtual environments. We have used three frequently used environment types and introduce a new “hybrid” environment in which real and virtual input is integrated, as a potentially viable alternative.

Traditionally, most navigation studies are carried out in the real world. Convenience is often a substantial factor in choosing a real world environment (Waller et al., 2004). Research in the real world poses a number of challenges. As opposed to studies in a laboratory setting, influencing and constraining a real world environment can be challenging. Enforcing identical conditions for all participants or specifically controlling the stimuli between participants to optimize task design is difficult to realize in real-world experiments. Potentially disturbing factors in the real world, like weather conditions, traffic, and noise, are difficult to control (Gillner and Mallot, 1998; Rey and Alcañiz, 2010). In the real world a topographical layout that is readily available can be used, or a layout has to be constructed, which can be time and money consuming. Moreover, participants with prior knowledge of the test environment add noise to measurements. However, in cases where locals are participating in the test, it can be difficult to avoid familiarity with the environment (Sandstrom et al., 1998).

Virtual reality technology is vastly increasing in quality and therefore being used more and more as a research tool (Maguire et al., 1999; Waller et al., 2003; Thiruvengada et al., 2011). In VR participants are placed into a three-dimensional, simulated environment with (partial) immersion. This environment can be manipulated by the user and is updated in real time (Azuma, 1997; Mills and Noyes, 1999; Schuemie et al., 2001; Bouvier et al., 2008). Virtual locomotion allows participants to move around in a virtual environment, while remaining in a restricted physical space (Templeman et al., 1999). Given these features VR can be a useful and versatile navigation research tool (Waller et al., 2004). Nowadays, virtual environments can be constructed with relative ease and it requires few resources to be able to run the necessary software (Mills and Noyes, 1999). The main advantage of virtual environments is that they can be modeled and controlled exactly to an experiment’s requirements, without having to build something similar in the real world (Sauzéon et al., 2011; Dombeck and Reiser, 2012). Even situations that would be impossible in the real world, such as teleportation, can easily be realized, which can be of great value in navigation research in particular. Secondarily, VR allows studies to be conducted in a lab setting, which means the conditions can be much further constrained and remain comparable for all participants. This high level of control improves the validity of the navigation studies (Schultheis et al., 2002). The lack of contextual factors however, makes a laboratory setting less realistic (Rey and Alcañiz, 2010). An additional advantage of VR is that it enables easy capture of precise data, for example a participant’s movement pattern over time. This means that research data becomes more readily available and is more precise than it would normally be (Rey and Alcañiz, 2010; Dombeck and Reiser, 2012). The advantages of VR imply that it can provide a reasonable alternative to traditional navigation research. For instance, VR has been used to analyze the *alignment effect* of You-Are-Here maps (Lukas et al., 2014); however, the use of VR itself may contribute to the effect (Montello, 2010).

The question remains whether the results of VR studies are valid in comparison with traditional navigation research methods and to what extent the results from a VR study equal performance in real life navigation. This question pertains to whether VR methods are suitable for navigation research or not. In the current study we address this question and introduce an alternative, which may overcome some of the disadvantages of traditional VR experiments.

A commonly used method to test navigation ability is to have participants move through an environment to familiarize themselves with a particular route through this environment. After such a learning phase, participants are asked to answer different types of questions in the testing phase, concerning their memory for landmarks, route properties, and the layout of the environment. This type of task relates to route learning in daily life; when we enter novel environments and try to find our way around, we also pay attention to various features of the environment and later attempt to recall these to retrace or return on our route. This approach will also be used in the current study: participants will study an environment in either a real or a virtual environment and are then tested on their knowledge of the environment. Those tests will focus on landmark, route, and survey knowledge (see e.g., Siegel and White, 1975; Montello, 1998), to cover a wide range of information required for successful navigation, reflecting properties of daily life navigation. Landmark knowledge reflects how well participants have memorized the identity and location of landmarks in the environment. Route knowledge concerns memory for route specific features of the environment, such as turns taken and order of landmarks along the route. Knowledge of the geometrical features of the environment irrespective of the route taken is reflected in survey knowledge.

In the comparison of real and virtual navigation two factors are prominent and are therefore included in the task design of the current experiment. If differences are found in navigation ability after studying a real or a virtual environment, this could well be due to the difference in locomotion between the conditions, as locomotion is present in typical experiments in a real environment and absent in most experiments using virtual input. As locomotion provides additional sensory input during learning (Klatzky et al., 1998; Rey and Alcañiz, 2010; Chrastil and Warren, 2013), it could be that performance increases when locomotion is part of the learning phase.

One particular element of locomotion is the directional information gained from interaction with the environment, as provided by vestibular input during motion and head rotation (e.g., McNaughton et al., 1995). It could be that differences in performance are related to the amount of directional information available and not just to the displacement of the participant. The current experiment therefore includes four conditions, in which not only performance after real and virtual learning is compared, but also the effects of both locomotion and directional information on performance. The virtual condition is most traditional: participants interact with a virtual environment shown on a computer screen, while remaining seated throughout the experiment. In this virtual condition locomotion and directional information are absent. The virtual+ condition is very similar, but only differs in the overt availability of directional information, by means of a compass shown during navigation. Locomotion is still absent, but directional information is available. In the real condition, participants walked around in a real environment and are asked to memorize what they encounter on their way. The hybrid condition is a novel way of using VR in navigation experiments. In this condition, participants walk around in the real world, while holding a portable digital device that provides all necessary navigational information in real time, by means of a GPS signal. In this condition both locomotion and directional information are available. This condition allows for flexible us of virtual elements, while maintaining the advantage of realistic locomotion in the real world. These four conditions allow for a comparison of performance after real and virtual learning and take into account locomotion and directional information. Given previous findings, we expect higher performance overall for the real condition. If this is because of locomotion, then performance on the real and hybrid conditions should be highly comparable and higher than performance on both the virtual and virtual+ conditions. If directional information is an important factor in itself, than performance on the virtual+ condition will be higher than on the virtual condition.

## Materials and Methods

### Participants

Seventy-eight participants (36 female, mean age = 21.7 years, SD = 2.4) performed the experiment, in exchange for course credit. All participants had normal or corrected to normal vision and signed informed consent prior to participation. Participants were randomly assigned to one of the four experimental conditions. The Santa barbara sense of direction scale (SBSDS) was used to match the resulting four groups based on self-reported spatial ability. The SBSDS has been shown to be a questionnaire for spatial ability with high reliability and internal consistency (Hegarty et al., 2002). The characteristics of each of the four groups are reported in Table 1.

**Table 1:** **Descriptives for all four participant groups**.

**Condition**	**N**	**%Male**	**Age (SD)**	**SBSODS score (SD)**
Real	19	36.8	21.9 (2.6)	63.1 (3.0)
Hybrid	20	60.0	23.2 (2.5)	64.9 (2.7)
VR	19	63.2	20.6 (1.7)	70.6 (2.5)
VR+	20	55.0	21.2 (2.1)	68.4 (3.0)

VR, virtual reality condition; VR +, virtual reality condition with directional information.

### Materials

The experiment consisted of route learning in four different conditions after which a series of route knowledge tests were administered. Participants studied a route in a real, hybrid, virtual, and virtual+ condition.

**FIGURE 1:**
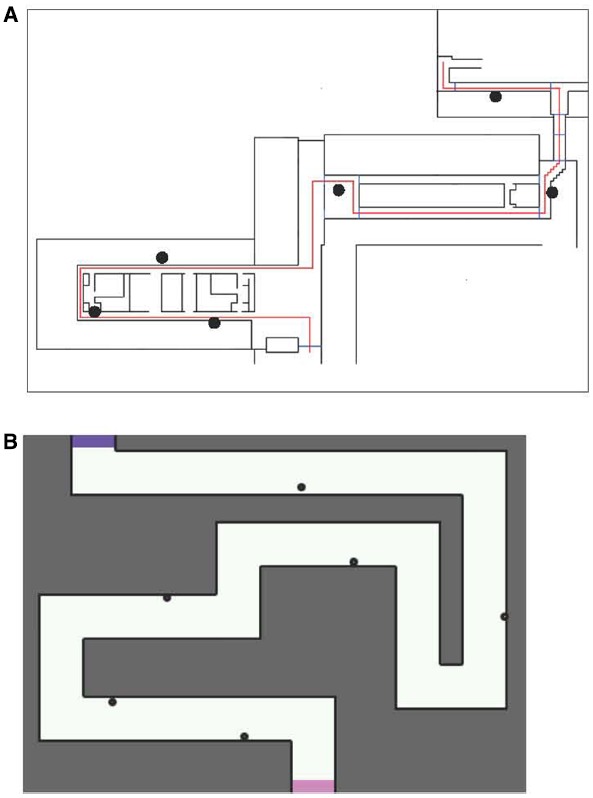
**Map of the routes used on the (A) real and (B) hybrid and virtual conditions.** In both maps, participants moved from the bottom to the top of the map. Dots indicate landmark positions.

In the real condition, participants walked along a route through three directly connected buildings on the campus of Utrecht University. Figure 1A illustrates the route. The route was approximately 260 m long and consisted of 10 turns. Some participants may have had previous exposure to a small section of the route, but none of them were familiar with this particular route. The experimenter walked with the participant to indicate what direction they should walk in.

**FIGURE 2:**
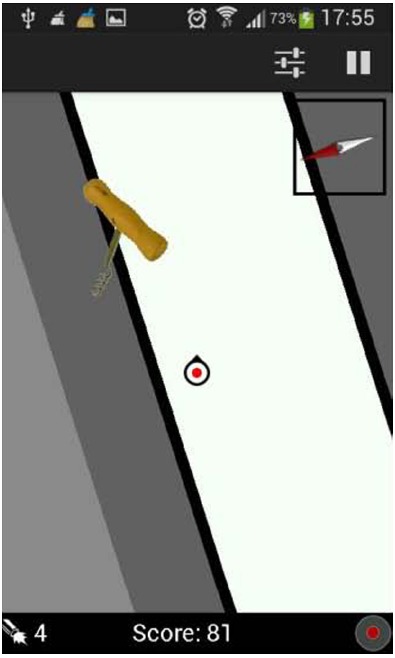
**Screenshot from the geoshooter application used in the hybrid condition**.

The hybrid condition combined a real and a virtual environment. Participants walked along the route on a hockey field, without any markings relevant to the route. Figure 1B illustrates the route. All route information was provided on a tablet computer, held by the participant. Due to real time GPS tracking, the route information was continuously updated to the participant’s position. The route selected, was chosen to match the route in the real condition as much as possible. It was 290 m long and consisted of 10 turns and 6 landmarks. For the digital display of the route, the Geoshooter application was used (Venselaar, 2014). This application was originally designed to allow game play in which players can “shoot” at virtual targets in physical space, while those objects are only presented digitally and not actually present in the real world. The application uses GPS and compass input from the mobile device (smart phone or tablet) carried by the player. A screenshot from the application is shown in Figure 2. Participants viewed their own position (a marker in the center of the screen) on a small portion of the total map, to have sufficient visual input to perform the task, while minimizing the amount of direct survey information depicted by the topview of the route. The map moved and rotated immediately and proportionally when the participant walked and rotated. A Samsung Galaxy Tab 1 was used, with a 10.1″ screen and a resolution of 1280 × 800 pixels.

**FIGURE 3:**
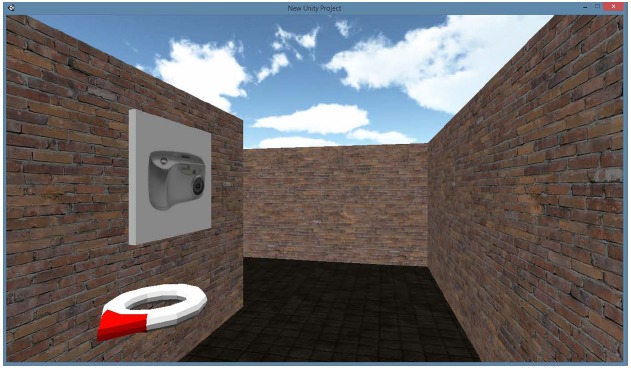
**Screenshot from the virtual + condition.** This image shows the virtual environment from the participant’s perspective with the directional information provided by the compass.

In the virtual condition, the same map as in the hybrid condition was used. The virtual environment was built in Unity 3D software (Unity Technologies, Los Angeles, CA, USA). Participants viewed the environment frontally as displayed on Figure 3. The route was depicted as a brick corridor without a ceiling, with sufficient contrast between the floor and walls. The scale of the environment resembled the real environment, with the camera positioned 1.75 m above the floor, at average eye height. Multiple light sources were used to avoid directional cues from shades. Movement speed was fixed to 5.5 km/h to resemble regular walking speed. Movement through the environment was controlled by the keyboard (moving forward) and the mouse (controlling direction), moving backward was not possible. The virtual environment was shown on a 15.6″ laptop with a resolution of 1366 × 768 pixels.

The *virtual+ condition* was identical to the virtual condition, with the exception of added direction information to the interface, as depicted in Figure 3. A floating compass was presented in the bottom left corner of the screen, with a red point continuously pointing to the “fictional north” of the environment.

In all conditions, six objects were used as landmarks; an apple, a battery, a photo camera, a plant, a torch, and a cork screw, selected from a standardized image set (Brodeur et al., 2010). These images were matched in factors like familiarity, visual complexity and viewpoint. In the real condition, the six landmarks relevant to the route knowledge tests were presented on A4 sheets of paper attached to the walls. In the digital conditions, digital images of these landmarks were placed along the route. In all conditions landmarks were positioned evenly along the route, three on the left side, three on the right side of the route. To ensure sufficient encoding of the landmarks and their spatial attributes, participants were required to interact with the landmarks. In the real condition, participants were asked to take a photograph of each of the landmarks when passing them. In the hybrid condition, they were asked to aim and shoot the landmarks. In the virtual conditions they were asked to take screen shots when they saw the landmarks along the route.

### Task Design and Procedure

The experiment was a between subjects study in which each of the four groups of participants was randomly assigned to one of the four route conditions. The hybrid and virtual conditions were preceded by a brief practice period, during which participants were familiarized with the controls and display of the device. In the study phase, participants were instructed to study the route as well as they could, without giving any indication of what type of questions would be asked afterward. After studying the route in the real, hybrid, virtual or virtual+ condition, route knowledge was tested by a series of tasks, presented on a laptop computer. In the real and hybrid conditions, participants had to walk to the testing location after completing the study phase. To control for the time between study phase and testing phase, participants were asked to count backward in intervals of 3 for 1 min, to ensure there was a 1-min pause between study and testing phase with the same cognitive load in all conditions. In the testing phase, five different tests were used.

1. In *landmark recognition* participants were shown 12 objects, 6 targets, and 6 distractors. For each object they were asked to indicate whether they had seen the object on the route or not. The scores indicate the percentage of correct responses.
2. Next, participants were asked to estimate *route distance* in meters. It was specifically mentioned that the virtual conditions were built to match regular dimensions in terms of wall height etc.
3. Participants then indicated *route position*. For each object they moved a horizontal slider to indicate where along the route they encountered the object. For example, if a landmark was present early on in the route, the participant should place the slider to the left side of the slider. The scores reflect the percentage of deviation from the correct position on the slider.
4. In the *pointing task*, participants were asked to point to the beginning and endpoint of the route, while imagining passing a particular landmark and facing in the forward route direction. An analog pointing device was used, which was placed horizontally on the table. The scores for this task reflect the mean deviation from the correct angle in degrees.
5. The last test concerned *map drawing* of the route. The participants were asked to draw the route they had walked and to indicate the position of the landmarks along the route. A maximum of 22 points could be obtained for this task.

## Results

First, to enable an overall comparison of all scores, the scores were normalized to z-scores. Then, a repeated measures general linear model (GLM) was performed, with task (landmark recognition, route distance, route position, pointing, map drawing) as within subjects factor and condition (real, virtual, virtual+, hybrid) as between subjects factor. This analysis revealed a significant interaction of task and condition, *F*(12,296) = 4.49, *p* < 0.001, partial η^2^ = 0.15. Main effects of task and condition did not reach significance. The interaction of task and condition calls for follow up analyses. For each task individually, the main effect of condition was examined by means of an ANOVA with condition as a between subject factor. In Table 2 all mean scores for all four groups are reported.

**Table 2:** **Mean performance for each task for each group of participants**.

**Task**	**Real**	**Hybrid**	**VR**	**VR+**
Landmark recognition	93.4 (0.11)	84.6 (12.3)	89.5 (13.3)	85.0 (14.2)
Route distance	0.50 (0.25)	0.53 (0.56)	1.04 (1.03)	0.74 (0.61)
Route position	12.4 (6.2)	13.4 (5.9)	13.2 (5.3)	14.7 (6.2)
Pointing	58.1 (25.3)	70.7 (22.3)	78.9 (20.4)	84.3 (16.3)
Map drawing	6.4 (5.2)	15.5 (3.6)	13.0 (2.4)	9.9 (3.3)

Landmark recognition is expressed in percentage correct, route distance in proportion of actual distance, route position in percentage deviation from actual position on slider, pointing in mean deviation in degrees, map drawing in score (range 0–22 points). Standard deviations in parentheses.

This ANOVA did not show a significant effect of condition for landmark recognition or route position (*p* > 0.05 in both cases). The main effect of condition approached significance in the route distance task, *F*(3,74) = 2.65, *p* = 0.055, partial η^2^ = 0.10. *Post hoc* analysis showed that for the route distance task none of the four conditions differed significantly from one another (*p* > 0.05 in all cases). The ANOVA showed a significant main effect of condition for both the pointing task, *F*(3,74) = 5.54, *p* < 0.01, partial η^2^ = 0.18, and the map drawing task, *F*(3,74) = 11.9, *p* < 0.001, partial η^2^ = 0.33. For the pointing task, Bonferroni corrected *post hoc* analyses showed that performance was higher for the real condition, as compared to the virtual (*p* < 0.05) and virtual+ (*p* < 0.01) conditions. For the drawing task, performance on the real task was higher, compared to the virtual (*p* < 0.05) and virtual+ conditions (*p* < 0.001). Furthermore, performance on the hybrid condition was significantly better than performance on the virtual+ condition (*p* < 0.001).

## Discussion

In this methodological study, we focused on different environments in which navigation ability can be measured. The main research question concerned the comparison of navigation performance in real versus virtual environments. Moreover, a novel condition was added in which real and virtual input were integrated. Traditionally, real environments are frequently used, but these have been shown to be costly and restricted in possible layouts and landmark options. Use of virtual environments is rapidly increasing as experimenters have full control over the layout and exterior of the environment, yet navigation in virtual environments is typically limited in physical involvement. Therefore, the current study compared performance in a real environment to a typical virtual condition (“virtual”). The isolated potential contribution of directional information was studied in the “virtual+” condition, which was very similar to the virtual condition. The only difference was the addition of a virtual compass during navigation, to aid in keeping track of the fictional “north” of the environment. Moreover, the potentially advantageous approach in which both real and virtual input is provided was used in the “hybrid” condition, in which participants actively moved through a virtual environment, presented on a portable tablet computer.

First of all, the significant interaction of task and condition along with the absence of a main effect of condition shows that the condition in which an environment was studied affects navigation performance, but differently across the different tasks. Learning condition did not affect performance on the landmark recognition task, the route position task, and the distance estimation task. This shows that landmark and route knowledge are not affected by the condition in which the environment was studied. In contrast, conditions did show different performance levels in the pointing task and the map-drawing task. Both these tasks typically rely on survey knowledge, or knowledge of the geometrical layout of the environment, extending beyond route knowledge. These results show that measures of navigation ability are identical for real and virtual measures, when landmark or route knowledge is measured. For survey knowledge, there are differences. In those measures, performance is highest for the real environment, and the virtual environments show the lowest performance. Locomotion therefore appears to contribute to better survey knowledge. The distinction between landmark, route, and survey knowledge is a prominent aspect of navigation research. They reflect cognitively distinctive features of navigation behavior (see e.g., Siegel and White, 1975; Montello, 1998). Landmark and route knowledge relate to navigational features that are learned on sight, all information is studied from an egocentric, or observer-based, perspective. For survey knowledge however, an allocentric, or environment-based perspective, is required, in which participants create mental maps of the environment (see e.g., Maguire et al., 2000). In such mental maps, positional information of different landmarks in relation to each other is reconstructed based on the sensory input of moving through the environment. It appears that this mental map creation is more successful when participants are exposed to the real and hybrid environments.

The contribution of directional information was studied with a virtual and a virtual+ condition, in which the only difference was the presence of a virtual compass. None of the tasks showed a difference in performance between these two conditions. Therefore directional information, as least as implemented in the current task design, does not contribute to navigation ability.

The current study is a first exploration of the cognitive properties of integrating real and virtual input in the hybrid condition. To this end, this condition was compared to performance in more traditional environments. As the results indicate, such a hybrid presentation may have substantial advantages over the traditional approaches. In future studies it is important to further enhance the current task design. The different environments should be created in such a way that they are fully identical. To further look into the issue of isolating locomotion from directional information, locomotion could also be added to a virtual environment, by means of a treadmill or head mounted device.

In short, it depends on the type of task at hand, which type of environment is suitable. For landmark and route knowledge, any of the options suffice, real, virtual, and the hybrid combination of both. If survey knowledge is studied, a real environment shows highest performance. Therefore, for survey knowledge tasks, real environments are suggested to be superior to virtual environments. However, the real condition does not significantly differ from the hybrid condition. Therefore, in case an experiment may benefit from virtual input, the hybrid condition may well be the best condition to use. The role of directional information as tested with the two virtual conditions seems to be very limited, as performance was not affected by whether direction information was present or not. We therefore suggest considering the novel hybrid condition, especially for survey knowledge and when external restrictions, such as layout or familiarity with a real environment, may hinder optimal use of a real environment.

### Conflict of Interest Statement

The authors declare that the research was conducted in the absence of any commercial or financial relationships that could be construed as a potential conflict of interest.
